# Imaging Challenges in the Diagnosis of Anatomical Variations of the Supra-Aortic Vessels: A Case Report and Review of Literature

**DOI:** 10.3390/diagnostics12010169

**Published:** 2022-01-12

**Authors:** Alexandra Dădârlat-Pop, Adrian Molnar, Alexandru Oprea, Raluca Tomoaia, Bianca Boros, Sorin Literat, Adela Serban, Simona Manole

**Affiliations:** 1 Cardiology Department, Heart Institute Niculae Stăncioiu, 19-21 Motilor Street, 400001 Cluj-Napoca, Romania; dadarlat.alexandra@yahoo.ro (A.D.-P.); biancaboros.93@gmail.com (B.B.); sorinl@yahoo.com (S.L.); adelamserban@yahoo.com; 2 Department of Cardiology, Iuliu Haţieganu University of Medicine and Pharmacy, 8 Victor Babes Street, 400012 Cluj-Napoca, Romania; 3 Cardiovascular Surgery Department, Heart Institute Niculae Stăncioiu, 19-21 Motilor Street, 400001 Cluj-Napoca, Romania; adimolnar45@yahoo.com (A.M.); alexandru_oprea2002@yahoo.com (A.O.); 4 Department of Cardiovascular Surgery, Iuliu Haţieganu University of Medicine and Pharmacy, 8 Victor Babes Street, 400012 Cluj-Napoca, Romania; 5 Department of Radiology and Medical Imaging, Heart Institute Niculae Stăncioiu, 19-21 Motilor Street, 400001 Cluj-Napoca, Romania; simona.manole@gmail.com; 6 Department of Radiology and Medical Imaging, “Iuliu Hatieganu” University of Medicine and Pharmacy Cluj Napoca, 8, Victor Babes, St, 400012 Cluj-Napoca, Romania

**Keywords:** hypoplastic internal carotid artery, compensatory intracerebral circulation, multimodality imaging, vertebral artery congenital variations

## Abstract

A 73-year-old woman was referred to our Cardiology Department due to recurrent headaches and dizziness. She had a history of hypertension of 10 years. In the territorial hospital, left internal carotid artery significant stenosis was suspected. Neurological examination and laboratory tests were normal. A neck vascular ultrasound was performed, showing a low bifurcation of the left common carotid artery (CCA) and a hypoplastic left internal carotid artery (ICA) with a sinuous path at the cervical level. Therefore, a computed tomographic (CT) angiography examination of the head and neck vessels was performed. The images confirmed the presence of a hypoplastic left ICA, anatomic variation in the left CCA, and also showed that the left vertebral artery (VA) was stemming directly from the aortic arch, exhibiting a kinking trajectory.

## 1. Introduction

Congenital anomalies of the cerebrovascular system are rare entities, with hypoplasia of the internal carotid artery (ICA) having an incidence lower than 0.01% [1]. Additionally, another congenital anomaly is the origin of the vertebral artery directly from the aortic arch, known as the “Adachi TYPE C” variation. These anomalies rarely coexist, due to embryological defects of development in the primitive aortic arch vessels [2]. Although all these congenital anomalies of the cervical vessels may remain asymptomatic, they are associated with important potential clinical implications. The diagnosis of these developmental abnormalities of the supra-aortic vessels is mainly based on imaging, such as ultrasonography, CT angiography (CTA), magnetic resonance angiography (MRA) or digital subtraction angiography (DSA). Congenital ICA hypoplasia is an uncommon disease, which may remain asymptomatic, or may be associated with non-specific symptoms. Although intracranial aneurysms may develop over time, there are no current guidelines regarding its management. Therefore, new data regarding ICA hypoplasia, its symptoms, other associated congenital vascular variations and follow-up strategies are needed in order to avoid cerebrovascular events.

## 2. Case Report

**Figure 1:**
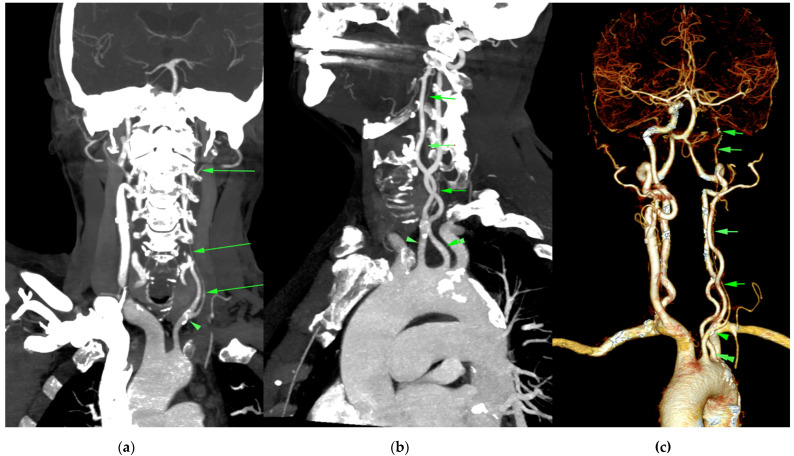
Cervicocerebral 3D CT angiography reconstruction: (**a**) MIP coronal; (**b**) MIP oblique sagittal; (**c**) VRT. The left CCA has a low-lying bifurcation (arrowhead) at approximately 2 cm from the aortic arch. The left ICA has a filiform trajectory (multiple green arrows). The left VA has an anomalous origin from the aortic arch (bold arrow). Compensatory hyperplasia of the contralateral CCA and ICA and both vertebral arteries.

**Figure 2:**
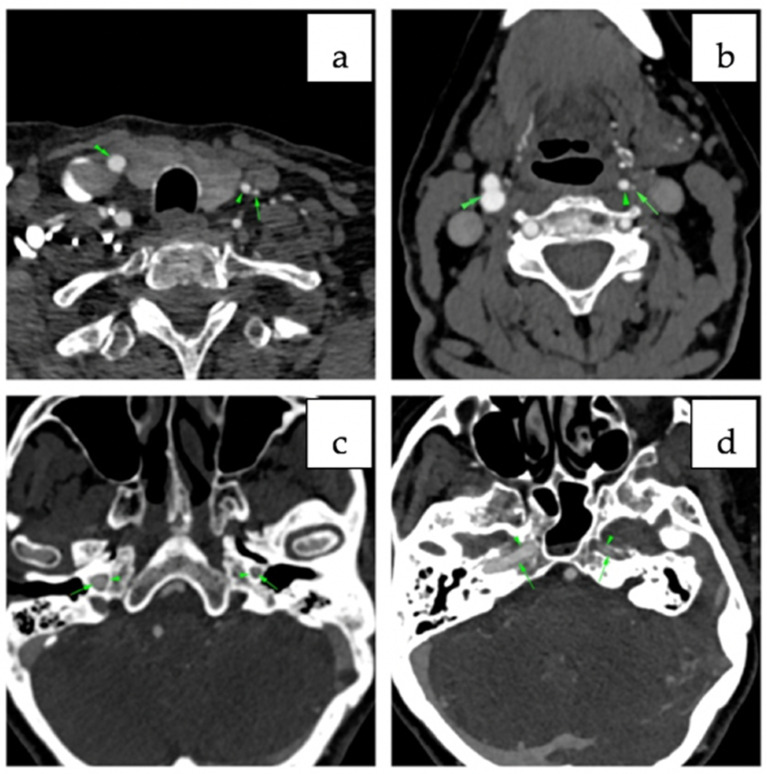
Axial cervical CT angiography: (**a**) in the lower third, near the thyroid—low bifurcation of the left carotid bulb, left ECA with normal lumen (arrowhead) and left ICA with filiform lumen (arrow); right ICA (double arrowhead); (**b**) in the middle third—normal bifurcation of the right carotid bulb (double arrowhead), left ICA with filiform lumen (arrow), left ECA with well-represented, normal lumen (arrowhead); (**c**,**d**) small diameter of the left carotid canal in the petrous temporal bone compared with the contralateral carotid canal (arrows).

**Figure 3:**
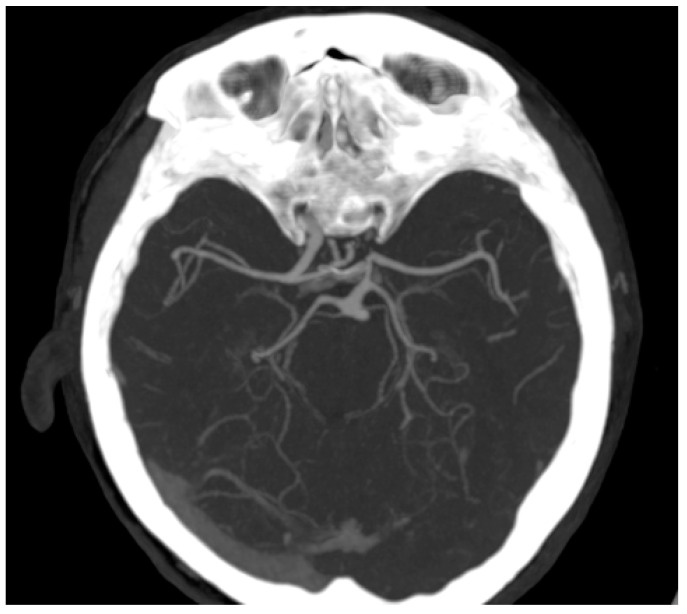
Cerebral CT angiography with MIP axial reconstruction: anatomic variation in the circle of Willis showing the absence of the A1 segment of the left ACA and right PCOM and compensatory well-represented left PCOM.

A 73-year-old woman was referred to our cardiology department for the treatment of a misdiagnosed left ICA stenosis based on a duplex ultrasonographic examination performed in an outer healthcare center in order to assess the underlying cause of her complaint of dizziness and drop attacks. We performed another extracranial duplex ultrasonography (US) of the cerebral vessels which actually showed a low bifurcation of the left CCA, whereas the left ICA was found to be hypoplastic, having a luminal diameter of 2.4 mm with reduced flow velocities. The left external carotid artery (ECA) had a diameter of 4 mm with a sinuous path at the cervical level, showing an increased diastolic blood flow. The contralateral CCA had a diameter of 7.8 mm with an atherosclerotic plaque at the bulb level without significant stenosis; the ICA was found to be distended with a diameter of 8.6 mm with increased flow velocities, and the ECA had a normal diameter and trajectory. On the basis of the US findings, a CT angiography examination of the head and neck vessels was performed. The images confirmed the presence of a hypoplastic left ICA 2 mm in diameter that could be followed up distally, with its diameter progressively reducing to 1 mm, as indicated in Figure 1 and Figure 2. Additionally, it certified the anatomic variation in the left CCA, which consisted of it having a low bifurcation at the level of the first thoracic vertebra (Figure 1). It was also noted that the ipsilateral anterior cerebral artery (ACA) was supplied from the anterior communicating artery (ACOM), whereas the ipsilateral medial cerebral artery was sustained predominantly by the posterior communicating artery (PCOM; Figure 3). Apart from the ICA hypoplasia, another interesting finding was that the left vertebral artery (VA) was not emerging from the left subclavian artery, but instead was stemming from the aortic arch, as its fourth branch. Additionally, the left VA had a slightly angulated trajectory at the level of C2 vertebra, which caused a stenosis of 63%. Therefore, treatment for the patient was conservative and regular follow-ups by non-invasive imaging were recommended in order to identify potential new aneurysms.

Written informed patient consent for publication has been obtained.

## 3. Discussion

Anomalies of the anatomy of the carotid–vertebral systems are complex and very rarely documented occurrences, frequently being accompanied by collateral circulation developments. Imagistic examinations play a key role in the diagnosis and understanding of extracranial and intracranial vessel blood flow, especially in pathological conditions, such as congenital anomalies. The diagnostic procedures in the detection of all those congenital anomalies start with vascular ultrasound (US). Therefore, pathological findings described by US examinations are further verified by CTA, MRA or DSA.

The complex anatomy of the carotid–vertebral arteries and the development of collateral circulation has been studied intensively for years. Aberrant variations in the carotid artery systems are uncommon findings and include hypo/aplasia of the internal carotid artery, anomalous cervical internal carotid artery branches, etc. [2,3,4,5,6,7,8,9,10,11].

The ICA is one of the most important arteries supplying the brain. Therefore, recognition of the congenital anomalies of the ICA is of special interest due to potential significant clinical implications, especially when endovascular procedures are considered. In normal embryogenesis, the ICA originates from the third arch artery and the dorsal aorta [3]. Hypoplasia of the internal carotid artery (HICA) is a rare condition, with an estimated prevalence of 0.01% [4]. HICA is more frequently unilateral and three times more common on the left side [4,5]. HICA was found in our patient on the left side as well. The mechanism that induces this anomaly has not yet been elucidated [6]. It has been hypothesized that the mechanism of HICA development may be the incomplete development of the fetal dorsal aorta [7].

Even though there are few cases described in the literature, the real incidence may be significantly underestimated because of the compensatory collateral circulation responsible for the paucity of symptoms. Therefore, the collateral circulation associated with HICA may have various pathways. In our case, the ipsilateral ACA was supplied through a patent ACOM, whereas the ipsilateral middle cerebral artery was supplied predominantly through the PCOM. This pathway of compensatory circulation associated with HICA is known as type 1 [8]. Even though these compensatory mechanisms are responsible for the leak of symptoms, patients with HICA deserve special consideration because of the risk of ACOM and PCOM aneurisms [9]. Cerebral aneurisms are more frequently found in HICA patients than in the general population (24–34% and 2–4%, respectively) [8]. Most often, HICA is diagnosed by US showing diffuse luminal narrowing of the internal carotid artery. However, it may be misdiagnosed with other conditions, such as carotid artery dissection, Takayasu disease, fibromuscular dysplasia or even with high-grade lesions, as in our case. Angio-CT or angio-RM should therefore be performed to confirm the diagnosis.

Anomalies of the vertebral arteries include variations in vertebral artery origin from the aorta or from the common carotid artery, hypoplasia/aplasia of one vertebral artery with compensatory dilatation of the contralateral artery, etc. [9,10,11]. Those anomalies are responsible for reduced anterograde blood flow, triggering numerous collateral compensatory vessel formation [10,11]. Usually, the left vertebral artery arises from the left subclavian artery medial to the thyrocervical trunk. Variations in the aortic arch are described in the literature; the condition when an anomalous left vertebral artery arises directly from the aortic arch is known as “Adachi TYPE C” variation [12,13]. Its prevalence is reported to be between 0.68% and 5.8%, having a female predominance [11]. Aberrant left vertebra are unlikely to cause any symptoms [12]. In addition to its aberrant origin, the left vertebral artery is ectatic and with a tortuous trajectory, known as dolichoectasia. Additionally, this variation is more commonly observed in women [14]. It may remain asymptomatic, but it may present with various clinical syndromes such as ischemic stroke, transient or permanent motor deficits, central sleep apnea, trigeminal neuralgia, or hydrocephalus [12,13,14]. Lusoria artery or aberrant right subclavian artery (ARSA) is another rare aortic arch branching anomaly, present in 0.4–1.8% of the general population. This implies a separate origin of the right subclavian artery directly from the aortic arch. Due to its trajectory near the esophagus, it may lead to dysphagia, known as dysphagia lusoria [15]. Therefore, the coexistence of multiple aortic arch branching anomalies, such ARSA, anomalous VA originating from the aortic arch, is a rare finding, and correct identification of these anatomical variations is essential before surgery or endovascular procedures.

There are no specific symptoms in the presence of anatomical anomalies of extra or intracranial artery congenital anomalies [6,7,8,9]; therefore, they may be under- or misdiagnosed. More often, they are found when other conditions of central nervous system vascularization impairment occur [4,7].

Due to the rarity of HICA cases, the management of patients with HICA is not well established. However, the treatment of diagnosed anatomical anomalies in adults is usually conservative. Due to the high risk of cerebral aneurisms, the management of cerebrovascular risk is mandatory. Therefore, strict control of arterial hypertension and of other cardiovascular risk factors is recommended, including lipid-lowering medication and quitting smoking. Additionally, urgent imagistic evaluation is indicated in patients with HICA when neurological symptoms appear. We strongly believe that a centralized registry with long-term follow-up data is mandatory in order to establish optimal management regimes of these patients.

## 4. Conclusions

HICA is a rare pathological condition, and associations with other congenital vascular anomalies, such as the “Adachi TYPE C” variation and vertebral dolichoectasia, are even rarer findings. These congenital anomalies are frequently associates with collateral compensatory circulation and anatomic variations in the circle of Willis. Therefore, reliable imagistic evaluations are critical for accurate diagnoses and follow-up. Initial evaluations with high-quality US, followed by CTA or MRA, are necessary in order to provide comprehensive anatomic information.
